# Frailty and Brain Myelin Across Adulthood: Multimodal MRI Insights From the BLSA

**DOI:** 10.1111/acel.70489

**Published:** 2026-04-14

**Authors:** Mustapha Bouhrara, Zhaoyuan Gong, Toshiko Tanaka, Luigi Ferrucci

**Affiliations:** ^1^ National Institute on Aging, NIH Baltimore Maryland USA

**Keywords:** aging, frailty, magnetic resonance imaging (MRI), myelin, white matter

## Abstract

Frailty is a state of reduced physiological resilience and increased vulnerability to adverse health outcomes, but its neurobiological mechanisms across the adult lifespan remain unclear. Emerging evidence suggests that white matter (WM) alterations may accompany frailty, but previous neuroimaging studies have focused mostly on older adults and used nonspecific MRI markers. This study investigates whether systemic frailty, quantified using a Frailty Index (FI), is associated with WM myelin content and integrity using advanced quantitative MRI. A total of 88 participants (aged 22–94 years, mean = 59.9 ± 20.0) from the Baltimore Longitudinal Study of Aging underwent multicomponent relaxometry MRI to measure myelin content, as well as relaxation rates R1 and R2 to probe white matter overall integrity. Multiple linear regression models examined the relationship between FI and whole‐brain and regional MRI biomarkers, adjusting for age and sex. Principal component analysis was used to explore global patterns of myelin and microstructural variation. Higher frailty scores were significantly associated with lower MWF across nearly all WM regions, especially in long‐range tracts such as the corona radiata and corpus callosum. R1 and R2 also showed inverse associations with FI, suggesting broader white matter vulnerability. These findings provide the initial evidence linking frailty to brain myelin alterations across the adult lifespan. The study highlights myelin degradation as a candidate neural substrate of frailty and underscores the importance of advanced quantitative MRI in detecting early brain vulnerability related to frailty. Further research is needed to clarify the causal relationship between frailty and myelin changes.

## Graphical abstract

Frailty, a marker of multisystem vulnerability, is associated with lower white matter myelin content across adulthood. Multicomponent MRI in the Baltimore Longitudinal Study of Aging revealed reduced myelin water fraction, particularly in callosal and projection fibers, suggesting myelin integrity as a neural correlate of systemic aging.

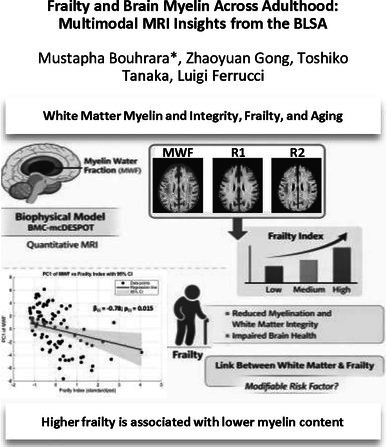

## 1 Introduction

Frailty is a multidimensional clinical syndrome characterized by the accumulation of age‐related health deficits across multiple physiological systems, leading to reduced homeostatic reserve and increased vulnerability to stressors. Unlike isolated disease or disability, frailty reflects systemic biological aging and diminished resilience, operationalized either through phenotype‐based criteria or deficit accumulation approaches such as the Frailty Index (FI) (Clegg et al. 2013; Vermeiren et al. 2016; Dedeyne et al. 2017). Although frailty is typically recognized in late life, its pathophysiology may originate from a systemic vulnerability that may affect the central nervous system (CNS) well before clinical symptoms appear (Siejka et al. 2022; Hunt et al. 2025; Sciancalepore et al. 2026). This possibility motivates efforts to define the neurobiological substrates of frailty, even in younger and middle‐aged adults, to identify early brain changes that precede overt frailty and age‐related decline.

Neuroimaging studies have begun to map the structural brain correlates of frailty, with early work implicating cerebral small vessel disease. Del Brutto and colleagues reported that frail and prefrail individuals exhibit higher white matter hyperintensity (WMH) burden (Del Brutto et al. 2017), and prospective data from the Tasmanian Study of Cognition and Gait showed that higher baseline WMH volume predicts frailty progression over ~4.4 years (Siejka et al. 2020). These findings suggest that macrovascular WM injury may contribute to the development of frailty rather than merely accompany it. Complementing these macrostructural associations, diffusion tensor imaging (DTI) studies provide evidence of microstructural alterations. Tian et al. found that frail older adults demonstrated higher mean diffusivity (MD) in frontal and subcortical gray‐matter regions and a trend toward reduced fractional anisotropy (FA) in the corpus callosum (Tian et al. 2020). Additional work indicates that frailty “subtypes” exhibit distinct WM patterns; for example, mobility frailty is associated with lower FA and higher MD in motor‐ and cognition‐related tracts such as the corpus callosum, whereas non‐mobility frailty corresponds to alterations in emotion‐related pathways including the cingulum and forceps major (Lin et al. 2025). Other cross‐sectional studies similarly report reduced WM integrity, including lower FA and higher MD and radial diffusivity (RD), in tracts such as the left cingulum and forceps major among frail individuals (Sumbul Sekerci et al. 2025). A recent systematic review reinforced these observations, highlighting consistent differences in WMH, gray‐matter volume, and DTI measures (e.g., lower FA, higher MD) between frail and non‐frail individuals (Harandi et al. 2025), findings supported by studies reporting greater WMH in frail older adults (Avila‐Funes et al. 2017; Kant et al. 2019). Longitudinal evidence further links microstructure to frailty risk: in the Atherosclerosis Risk in Communities Neurocognitive Study, lower FA and higher MD, along with WMH burden, predicted subsequent frailty (Ducca et al. 2023), and Maltais et al. reported similar prospective associations (Maltais et al. 2020). Frailty has also been associated with gray‐matter atrophy; in a large Japanese cohort, features such as slowness and weakness were related to reduced volume in regions including the hippocampus, amygdala, orbitofrontal and medial prefrontal cortex, inferior frontal gyrus, and insula (Nishita et al. 2019). Together, these findings indicate that the neural correlates of frailty span macrostructural vascular injury, WM microstructural compromise, and regionally specific gray‐matter vulnerability.

Beyond microstructure, some neuroimaging research has begun to explore interactions between frailty and other clinical phenotypes. For instance, a pilot study comparing older adults with both late‐life depression and frailty to non‐frail/non‐depressed controls found significant abnormalities in WM tracts associated with affective and structural brain changes (Shuster et al. 2023). Such findings suggest that comorbid conditions may exacerbate neural vulnerability and highlight the importance of considering multidimensional frailty phenotypes.

While prior neuroimaging studies have linked frailty to global WM abnormalities, these measures provide limited insight into the specific biological substrates involved. WMHI primarily reflect macrovascular injury, and DTI‐based metrics capture composite microstructural features without isolating myelin‐specific processes. In contrast, myelin integrity is directly relevant to neural conduction velocity, motor coordination, and processing speed (Gong et al. 2023; Gong, Bilgel, et al. 2024; Gong, Faulkner, et al. 2024); functions central to frailty phenotypes such as slowness and weakness. Moreover, oligodendrocytes are particularly sensitive to systemic inflammation, metabolic dysregulation, and vascular compromise—biological pathways strongly implicated in frailty (Zhang et al. 2026). Thus, myelin degeneration may represent a mechanistically plausible interface linking multisystem physiological vulnerability with CNS dysfunction. Such markers may also help identify neural substrates that are sensitive to interventions targeting systemic resilience.

Despite this biological relevance, the contribution of myelin to frailty‐related neural vulnerability remains largely unexplored. Although myelin disruption is a well‐established feature of aging and neurodegenerative diseases (Faulkner, Gong, Bilgel, et al. 2024; Faulkner, Gong, Guo, et al. 2024), its specific relationship with frailty across adulthood is unknown. To address this gap, we examined associations between a multidimensional FI and myelin‐sensitive MRI biomarkers in adults aged 22–94 years from the Baltimore Longitudinal Study of Aging (BLSA) (Ferrucci 2008). We employed quantitative MRI techniques, including myelin water fraction (MWF) and relaxation rates (R1, R2). MWF provides a myelin‐specific estimate (Laule et al. 2006, 2008; Bae et al. 2026), while the relaxation rates R1 and R2 offer complementary information to myelin‐specific MWF while retaining biological relevance to WM integrity (Deoni 2010). Specifically, R1 and R2 are derived from SPGR and bSSFP (steady‐state) sequences with very short echo‐times (TE), which make them highly sensitive to the fast‐relaxing water pool trapped between myelin bilayers. R1 primarily reflects macromolecular content and tissue composition, including lipids in myelin. R2 captures interactions between water and its microenvironment and is sensitive to multiple microstructural properties, including myelin content, axonal density and integrity, iron content, and tissue hydration. Thus, while R1 and R2 are not strictly myelin‐specific, they are heavily influenced by the myelin water pool and other relevant tissue characteristics, providing a broader assessment of WM microstructural health (Callaghan 1991). In combination with MWF, these metrics allow us to distinguish myelin‐specific alterations from more generalized microstructural changes, enhancing the biological interpretability of frailty‐related WM differences. Based on the biological vulnerability of late‐myelinating and association WM pathways to aging and systemic stressors, we hypothesized that greater frailty would be associated with lower myelin content, particularly in frontal and motor‐related regions implicated in slowness and weakness. We further examined whether these associations varied across the adult lifespan. Complementary microstructural measures (R1 and R2) were expected to show convergent patterns, reflecting broader WM tissue changes including in myelination. By combining region‐specific analyses with principal component analysis across imaging modalities, our study captures both localized and system‐level patterns of WM myelination, providing a more comprehensive understanding of how frailty relates to myelin and WM integrity through adult lifespan.

## 2 Methods

### 2.1 Participants

Participants were drawn from the BLSA study (Ferrucci 2008), an ongoing prospective cohort designed to characterize normative and pathological aging trajectories in Baltimore‐DC area. All participants underwent detailed clinical, cognitive, and physiological assessments at each study visit at the National Institute on Aging (NIA) Clinical Research Unit in Baltimore, MD. Eligibility for MRI scanning followed strict exclusion criteria shared across BLSA imaging protocols, including the absence of major neurological disease (e.g., dementia, stroke, Parkinson's disease, and epilepsy), major psychiatric illness, clinically significant cardiovascular or pulmonary disease, and metastatic cancer. Individuals with metal implants, claustrophobia, or contraindications for MRI were excluded from imaging. The BLSA protocol and all study procedures were approved by the Institutional Review Board of the National Institutes of Health (NIH), and all participants provided written informed consent at each visit.

### 2.2 Frailty Index (FI)

In this study, frailty status was determined using FI, a method that quantifies an individual's level of frailty based on the accumulation of health deficits, including symptoms, signs, diseases, and disabilities (Mitnitski et al. 2001). FI is calculated as the ratio of the number of deficits present to the total number of deficits considered. The details of the FI assessment in the BLSA are provided elsewhere (Tanaka et al. 2022). Briefly, the FI was computed using 44 variables. These included self‐reported difficulty with basic and instrumental activities of daily living, self‐rated health, items related to depressive symptoms, cognitive function, the prevalence of common age‐related conditions, 5% weight loss in the past year, low physical activity, slowness, and weakness. The final index is a ratio of the number of deficits to the total number of deficits, ranging from 0 to 1, with higher values indicating a greater degree of frailty.

### 2.3 MRI Data Acquisition

All participants underwent a quantitative imaging protocol consisting of multicomponent relaxometry based on the Bayesian Monte‐Carlo multicomponent driven equilibrium single pulse observation of T_1_ and T_2_ (BMC‐mcDESPOT) method for whole‐brain mapping of myelin water fraction (MWF), longitudinal relaxation rate (R1), and transverse relaxation rate (R2) (Bouhrara and Spencer 2015, 2016, 2017). The protocol incorporated 3D spoiled gradient recalled echo (SPGR) and 3D balanced steady‐state free precession (bSSFP) acquisitions. SPGR images were acquired with flip angles (FAs) of [2 4 6 8 10 12 14 16 18 20]°, echo time (TE) of 1.37 ms, repetition time (TR) of 5 ms, and an acquisition time of approximately 5 min. bSSFP images were obtained with FAs of [2 4 7 11 16 24 32 40 50 60]°, TE of 2.8 ms, TR of 5.8 ms, and an acquisition time of approximately 6 min per phase increment. To correct off‐resonance banding, bSSFP data were acquired using radiofrequency phase increments of 0° or 180°, yielding a total bSSFP scan time of ~12 min. All SPGR and bSSFP images were acquired with a matrix size of 150 × 130 × 94 and isotropic voxel size of 1.6 mm^3^, and reconstructed to 1 mm^3^ isotropic resolution. A double‐angle method (DAM) B1 mapping protocol was collected using two fast spin‐echo images with flip angles of 45° and 90°, TE of 102 ms, TR of 3000 ms, and voxel size of 2.6 × 2.6 × 4 mm (acquisition time ~4 min) (Bouhrara and Spencer 2018). The resulting low‐resolution B_1_ map was interpolated to match the SPGR/bSSFP grid. The total MRI acquisition time was approximately 21 min. All scans were performed on a 3T Philips Achieva MRI scanner (Philips Healthcare, Best, The Netherlands) using the built‐in body coil for transmission and an 8‐channel phased‐array head coil for reception.

### 2.4 MRI Data Processing

Preprocessing followed established BMC–mcDESPOT procedures. Using FSL's Linear Image Registration Tool (Jenkinson et al. 2012), all SPGR, bSSFP, and DAM images were rigidly aligned to the SPGR acquisition acquired at an 8° flip angle. Whole‐brain MWF maps were generated from the co‐registered SPGR, bSSFP, and B_1_ data sets using the Bayesian implementation of mcDESPOT (Bouhrara and Spencer 2015, 2016, 2017). The model accounted for a two‐component T_2_ distribution, representing a short‐T_2_ myelin‐associated water pool and a long‐T_2_ intra‐/extracellular water pool, and incorporated corrections for nonzero TE (Bouhrara and Spencer 2015). Whole‐brain R1 maps were generated using DESPOT1 from SPGR and B_1_‐corrected data, and whole‐brain R2 maps were computed using DESPOT2 from bSSFP and DAM data (Deoni et al. 2005; Deoni 2009). R1 and R2 reflect microstructural tissue characteristics related to water mobility, macromolecular content, and myelin‐associated lipids (Deoni 2010), providing complementary indices of WM tissue integrity.

An averaged SPGR image across flip angles was nonlinearly registered to MNI152 standard space using FSL. The resulting transformation was applied to the MWF, R1, and R2 maps. Eleven WM regions of interest (ROIs), including whole brain (WB), frontal lobes (FL), temporal lobes (TL), parietal lobes (PL), occipital lobes (OL), corona radiata (CR), corpus callosum (CC), longitudinal fasciculus (LF), fronto‐occipital fasciculus (FOF), internal capsule (IC), and forceps (For), were defined based on probabilistic WM atlases and eroded to minimize partial‐volume effects. Regional mean values of MWF, R1, and R2 were extracted for all ROIs.

### 2.5 Statistical Analysis

Associations between the FI and MRI (MWF, R1, or R2) metrics were evaluated using multiple linear regression. Each model included MWF, R1, or R2 as a dependent variable, with FI as the predictor of interest and age and sex included as covariates. The regression model is given by: $\mathrm{MRI}=\beta_{0}+\beta_{\mathrm{FI}}\mathrm{FI}+\beta_{\mathrm{age}}\mathrm{age}+\beta_{\mathrm{sex}}\mathrm{sex}+\epsilon .$ All continuous variables were z‐scored prior to analysis. A sensitivity analysis was also conducted excluding the cognitively impaired participants.

To quantify system‐level patterns of myelin and WM microstructure, we applied principal component analysis (PCA) to the ROI‐specific measures for each MRI metric (MWF, R1, and R2) separately. For each participant, PC1 captures the dominant pattern of variance across all ROIs, reflecting regions that tend to vary together in myelin content or WM microstructure. Using PC1 allows us to summarize brain‐wide trends in myelin and microstructural integrity while preserving the specificity of MWF for myelin and the complementary sensitivity of R1 and R2 to microstructural features. PC1 scores were then used as dependent variables in regression models with the FI as the predictor, controlling for age and sex.

To examine whether the association between FI and MRI metrics differed across adulthood, we tested interaction terms between age and FI for each MRI metric (MWF, R1, and R2).

To evaluate potential nonlinear associations between FI and MRI, we additionally included a quadratic term for the Frailty Index (FI^2^) in all regression models (ROI‐wise and PCA‐PC1 analyses), with age and sex as covariates. This allowed us to test whether the relationship between frailty and myelin‐sensitive MRI metrics (MWF, R1, and R2) deviated from linearity across the adult lifespan.

ROI‐wise analyses were FDR‐corrected across regions within each MRI biomarker. Statistical significance was defined as *p* ≤ 0.05. All statistical analyses were performed using MATLAB R2025a (MathWorks, Natick, MA, USA).

## 3 Results

A total of 88 participants, with available BMC‐mcDESPOT MRI scans and FI measures from the BLSA were included in the analysis of whom 11 were cognitively impaired (4 with dementia and 7 with mild cognitive impairment [MCI]). Ages ranged from 22 to 94 years, with a mean age of 59.9 years (SD = 20). The sample included both men and women, with a relatively balanced sex distribution (48 females, 40 males). FI values varied widely, spanning 0.005–0.41, indicating substantial variability in accumulated health deficits. The mean FI was 0.1 (SD = 0.08), consistent with a cohort exhibiting generally low to moderate frailty. Together, these demographic characteristics demonstrate a heterogeneous sample suitable for examining associations between frailty, age, sex, and myelin‐related MRI biomarkers.

**FIGURE 1:**
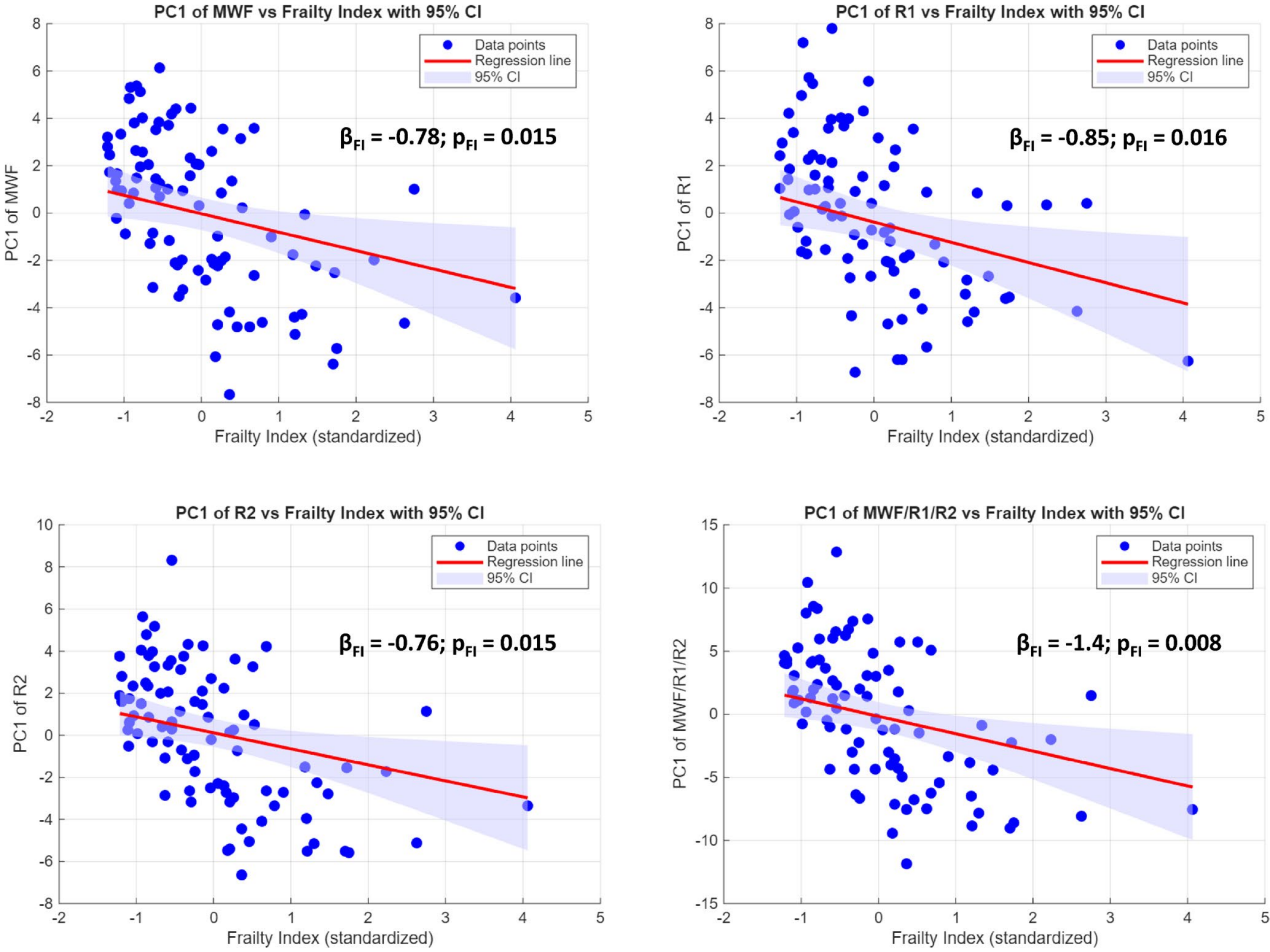
Association between frailty and white matter (WM) MRI biomarkers. Scatterplots show the first principal component (PC1) derived from regional MRI metrics plotted as a function of the frailty index (FI), with age and sex included as covariates in the multiple linear regression models. PCA was applied in two ways: (1) metric‐specific PCA separately for MWF, R1, or R2 across 11 WM ROIs (frontal, parietal, temporal, occipital, and select association tracts; see Figure 2 for full ROI list), where PC1 captures the dominant pattern of coordinated variation in myelin content (MWF) or myelin‐sensitive WM integrity (R1 and R2) across regions; and (2) combined PCA across all MRI metrics and ROIs together, where PC1 summarizes system‐level patterns of variation across both myelin‐specific and myelin‐sensitive measures. Similar trends across MWF, R1, and R2 reflect shared sensitivity to underlying WM myelin and microstructure and global covariance captured by PC1, rather than equivalence of these metrics. Regression coefficients and *p* values for FI are displayed on each panel.

Figure 1 illustrates the associations between frailty and myelin‐sensitive MRI biomarkers. Scatterplots show the PC1 derived from regional MWF, R1, or R2 values, or from all MRI biomarkers combined, plotted against the FI. Age and sex were included as covariates in the multiple linear regression models. MWF, a direct measure of myelin content, was inversely associated with frailty, indicating lower myelin levels in participants with higher FI. R1 and R2, which are sensitive to myelin and other WM microstructural tissue changes, showed similar trends, reflecting broader microstructural alterations associated with frailty. Standardized regression coefficients (β) and corresponding *p* values for FI are displayed on each panel, highlighting the strength and significance of these associations. Sensitivity analyses excluding the four participants with the highest frailty index (*z*‐scored FI > 2) yielded slightly steeper slopes and increased significance, indicating that extreme FI values influence but do not change the direction of associations. Although MWF, R1, and R2 show visually similar associations with frailty in the scatterplots, this similarity reflects shared sensitivity to underlying WM microstructure rather than redundancy between measures. Specifically, MWF provides a more myelin‐specific estimate, whereas R1 and R2 capture broader microstructural properties that are nonetheless strongly influenced by myelin content. In addition, the use of PCA emphasizes global covariance patterns across regions, such that PC1 captures common variance shared across WM tracts. As a result, similar trends across modalities are expected when a global biological factor, such as frailty, affects multiple aspects of WM structure.

**FIGURE 2:**
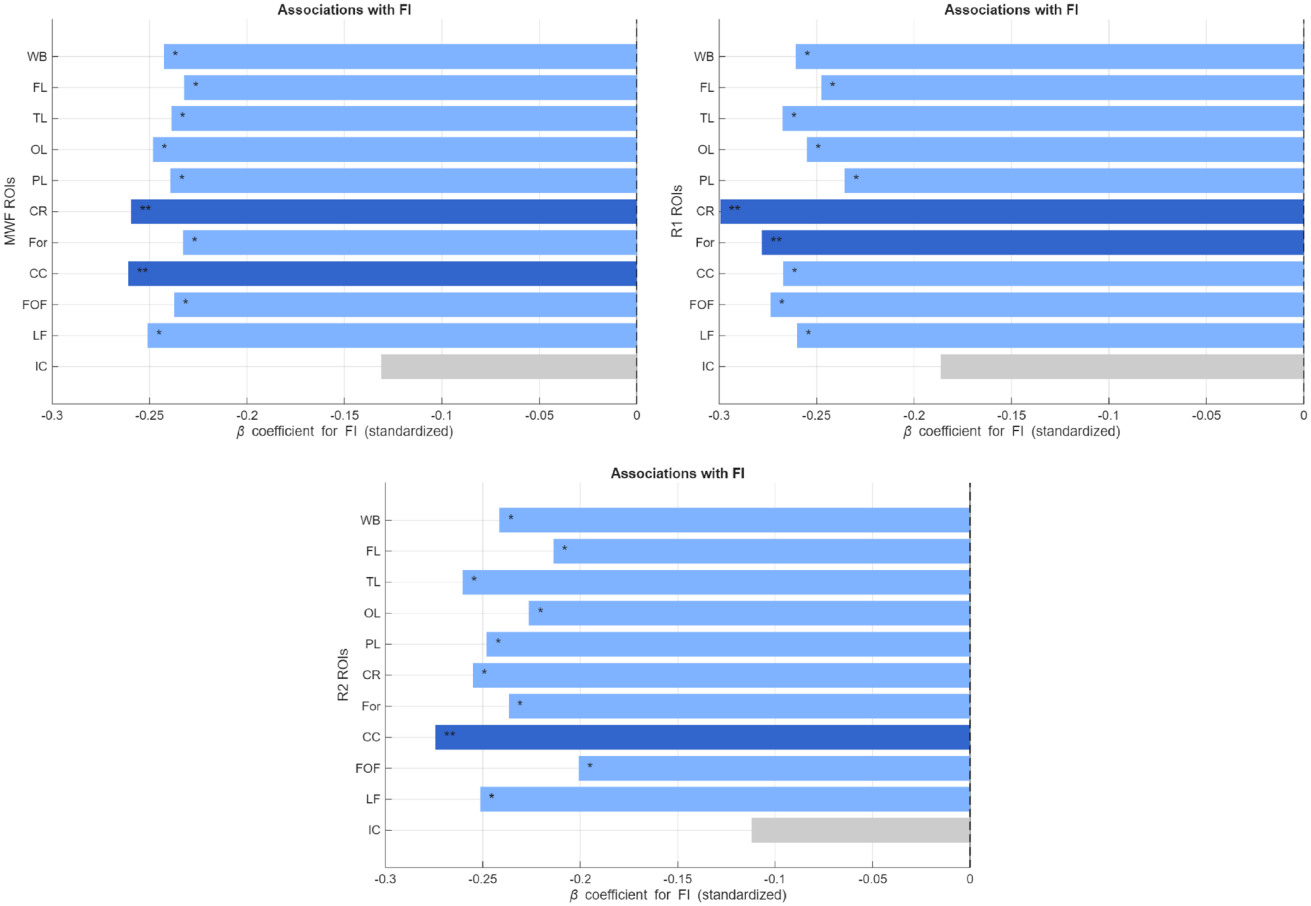
ROI‐wise standardized regression coefficients (β_FI_) for the associations between Frailty Index (FI) and regional MRI biomarkers (i.e., MWF—top left, R1—top right, or R2—bottom), controlling for age and sex. *Indicates FDR‐corrected *p* < 0.05 (light blue bars), and **indicates FDR‐corrected *p* < 0.01 (dark blue bars). Gray bars indicate nonsignificant associations. Full ROI‐wise results are reported in Figure 3A. CC, corpus callosum; CR, corona radiata; FL, frontal lobes; FOF, fronto‐occipital fasciculus; For, forceps; IC, internal capsule; LF, longitudinal fasciculus; OL, occipital lobes; PL, parietal lobes; TL, temporal lobes; WB, whole brain.

**FIGURE 3:**
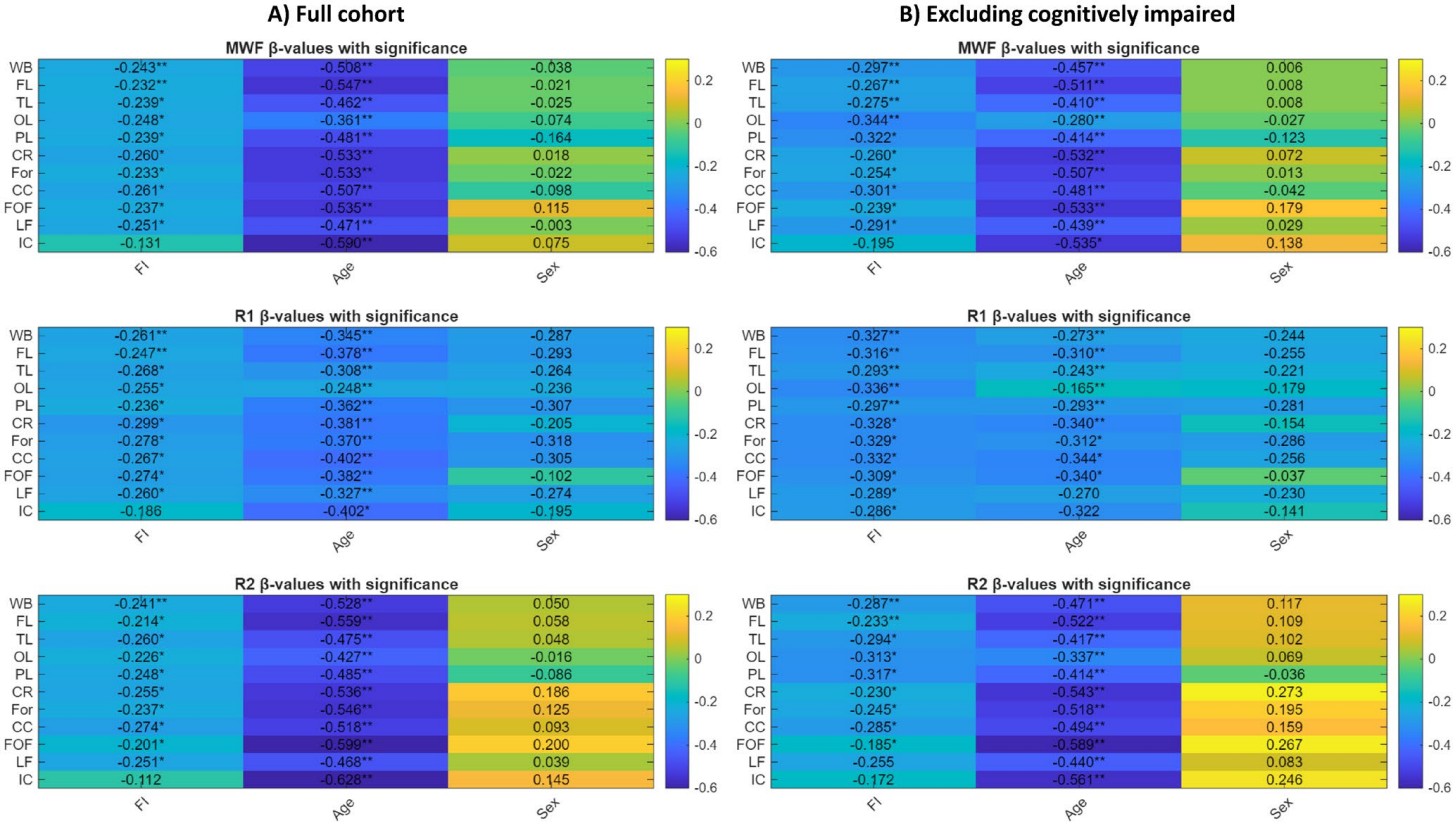
ROI‐wise standardized regression coefficients (β) and *p* values, after FDR correction, for associations between frailty index (FI), age, and sex with regional MRI biomarkers (MWF, R1, and R2) are shown for (A) the full cohort and (B) excluding cognitively impaired participants. Negative β_FI_ values indicate lower myelin content or reduced white matter (WM) integrity with higher frailty. CC, corpus callosum; CR, corona radiata; FL, frontal lobes; FOF, fronto‐occipital fasciculus; For, forceps; IC, internal capsule; LF, longitudinal fasciculus; OL, occipital lobes; PL, parietal lobes; TL, temporal lobes; WB, whole brain; *Indicates *p* < 0.05; **indicates *p* < 0.01.

Figure 2 shows ROI‐wise standardized regression coefficients (β_FI_) for the associations between FI and regional MRI biomarkers (MWF, R1, and R2), with age and sex included as covariates. MWF demonstrated significant negative associations with FI across nearly all regions, except for the internal capsules (IC), indicating that higher frailty is associated with lower myelin content (Figure 2A). The strongest effects were observed in the corona radiata (CR) [β_FI_ = −0.260; *p*_FI_ = 0.018] and corpus callosum (CC) [β_FI_ = −0.261; *p*_FI_ = 0.019], followed by the longitudinal fasciculus (LF) [β_FI_ = −0.251; *p*_FI_ = 0.031]. R1 showed a similar pattern to MWF, with the largest effect in the CR [β_FI_ = −0.299; *p*_FI_ = 0.020], indicating that more severe frailty is associated with lower WM integrity. R2 exhibited comparable trends, with the highest effect sizes found in the CC [β_FI_ = −0.274; *p*_FI_ = 0.031], followed by the temporal lobes (TL) [β_FI_ = −0.260; *p*_FI_ = 0.013] and CR [β_FI_ = −0.255; *p*_FI_ = 0.017]. Across all metrics, higher age was associated with lower myelin content and WM integrity (*p*_age_ < 0.05). Full ROI‐wise regression results, including β coefficients and FDR‐corrected *p*‐values, are reported in Figure 3A.

Across all MRI metrics and PCA PC1, the FI^2^ term was not significant, indicating that the relationship between frailty and myelin/myelin‐sensitive WM measures is approximately linear. This suggests that myelin content and WM microstructural integrity decline proportionally with increasing frailty, without evidence of acceleration or plateau effects across the range of FI observed in our sample. To determine whether frailty‐related differences in myelin and WM microstructure varied with age, we tested age × FI interactions for each MRI metric (MWF, R1, and R2). No significant interactions were observed (all *p* > 0.1), suggesting that the associations between frailty and myelin or WM integrity are largely stable across the adult lifespan in this cohort.

After excluding the 11 participants with dementia or MCI (Figure 3B), the associations between FI and all MRI biomarkers remained significant and broadly consistent with the full‐sample results (Figure 3A), however, the regional pattern of effect sizes shifted. For MWF, all 10 ROIs retained the significant inverse associations, with the strongest associations observed in the occipital lobe (OL) [β_FI_ = −0.344; *p*_FI_ = 0.007] and parietal lobe (PL) [β_FI_ = −0.322; *p*_FI_ = 0.010], followed by the corpus callosum (CC) [β_FI_ = −0.301; *p*_FI_ = 0.019]. These regions exhibited larger effects compared with the full sample, where the corona radiata (CR) and CC had been most prominent. For R1, all regions, including the internal capsules (IC), were significant, with the largest effects found in the CC [β_FI_ = −0.332; *p*_FI_ = 0.015], forceps (For) region [β_FI_ = −0.329; *p*_FI_ = 0.011], and CR [β_FI_ = −0.328; *p*_FI_ = 0.010], indicating similar convergence of frailty‐related microstructural vulnerability across major WM pathways. For R2, the longitudinal fasciculus (LF) lost significance, with the strongest association observed in the parietal lobe (PL) [β_FI_ = −0.317; *p*_FI_ = 0.012], followed by the OL [β_FI_ = −0.313; *p*_FI_ = 0.011], and the CC [β_FI_ = −0.285; *p*_FI_ = 0.031]. Whole‐brain R2 also remained significant [β_FI_ = −0.287; *p*_FI_ = 0.005]. Age was significantly associated with all MRI biomarkers, with standardized effect sizes roughly twice as large as those of frailty. FI values did not significantly differ between cognitively impaired and cognitively normal participants after adjustment for age and sex (*p* = 0.12). Given the small number of CI participants (*n* = 11), this analysis should be interpreted cautiously. Together, these results indicate that the FI‐brain myelin and microstructure relationships are robust to the exclusion of cognitively impaired individuals, although the specific regions showing the largest effects differ from the full‐sample analysis. Notably, occipital and parietal regions emerged as stronger contributors for MWF and R2 in the cognitively normal subgroup (Figure 3A), whereas the CR and CC remained consistently vulnerable structures across all metrics (Figure 3). Full ROI‐wise results are reported in Figure 3B.

## 4 Discussion

### 4.1 Summary of Findings

In this study, we provide evidence linking systemic frailty, defined by FI, to lower cerebral myelin content (via MWF MRI) and broader WM vulnerability (via R1 and R2 MRI) across the adult lifespan. Although participants from the BLSA are generally healthier and less frail than community‐based cohorts, we still observed robust inverse associations between FI and myelin/WM integrity, underscoring the sensitivity of our MRI biomarkers of myelin to detect early brain vulnerability even in a relatively resilient cohort. While age remained the dominant predictor across all MRI biomarkers, FI showed independent moderate associations with myelin and microstructural measures, indicating that frailty captures biological vulnerability beyond chronological aging alone. These findings are consistent with the well‐established impact of age on cerebral tissue (Kiely et al. 2022) and the progressive increase in frailty with chronological age observed across populations (Mitnitski et al. 2002; Stolz et al. 2019). The persistence of FI effects in both the full sample and the cognitively normal subgroup further supports the conclusion that frailty contributes uniquely to myelin and WM alterations. Importantly, the consistency of these associations across multiple imaging modalities (MWF, R1, and R2), as well as their persistence after excluding cognitively impaired participants, supports the robustness of the observed relationships despite differences in MRI signal model assumptions and measurement sensitivities.

### 4.2 Relation to Prior Frailty‐Neuroimaging Work

Our findings are broadly consistent with prior work showing that frailty is associated with WMH burden, gray‐matter atrophy, and DTI‐based microstructural WM alterations, as described above in the Section 1. However, these earlier studies relied on diffusion metrics or lesion burden, which lack specificity for myelin. Although neuroimaging work has not directly assessed myelin, molecular studies support a multisystem view of frailty; for example, a plasma proteomic FI based on a 25‐protein signature predicts mortality and dementia, highlighting frailty as not only a clinical or functional syndrome but a molecular one (Sathyan et al. 2025). Together, these observations underscore the need for tissue‐specific neural markers that can link systemic dysregulation to brain vulnerability.

Systemic frailty is characterized by chronic low‐grade inflammation, metabolic dysregulation, vascular compromise, and diminished physiological reserve; these are biological processes that are known to adversely affect oligodendrocyte function and myelin maintenance (Nasrabady et al. 2018; Dimovasili et al. 2023). Experimental and human studies demonstrate that myelin is particularly vulnerable to inflammatory cytokines, oxidative stress, impaired glucose metabolism, and reduced cerebral perfusion (Bouhrara et al. 2020, 2022; Kiely et al. 2023; Schlett et al. 2023; Faulkner, Gong, Bilgel, et al. 2024; Faulkner, Gong, Guo, et al. 2024; Shao et al. 2026), all of which are frequently observed in frail individuals. Because myelin integrity is essential for maintaining conduction velocity, network synchrony and efficient communication across distributed neural systems, its disruption may represent a biologically plausible pathway through which multisystem physiological vulnerability manifests as slowness, weakness, and reduced resilience.

Our study addresses this gap by providing the first direct evidence that frailty is associated with myelin‐sensitive MRI biomarkers across the adult lifespan. Higher frailty was linked to lower myelin content (MWF) and complementary alterations in WM microstructure (R1 and R2), both globally and across major WM pathways, independent of age and sex. These associations persisted in cognitively normal adults, suggesting that frailty‐related myelin changes emerge before overt cognitive decline and may reflect an early dimension of brain aging. By integrating myelin‐specific and microstructural measures, our results identify myelin integrity as a potential neural substrate of frailty and offer a more biologically grounded framework for understanding how systemic vulnerability manifests in the brain.

### 4.3 Regional WM Vulnerability in Frailty

We observed the largest standardized associations between FI and MRI biomarkers in the corpus callosum (CC) and corona radiata (CR), although the precise regional pattern varied across metrics and shifted when cognitively impaired participants were excluded. The CC, as the brain's principal commissural tract, contains a dense array of heavily myelinated fibers connecting homologous cortical regions. Its central integrative role and high myelin content make it especially sensitive to systemic physiological vulnerability, such that even modest myelin loss yields relatively large changes in myelin‐sensitive MRI biomarkers. The CR, a major projection system containing ascending and descending fibers, is highly metabolically active and energy demanding and, therefore, it is particularly susceptible to microvascular compromise, inflammation, and metabolic stress; these biological pathways are strongly implicated in frailty.

After excluding individuals with dementia or MCI, the pattern of strongest effects shifted: the occipital (OL) and parietal (PL) lobes showed the largest MWF associations, whereas for R1 the strongest effects were observed in the CC, the forceps, and the CR. For R2, the PL and OL exhibited the largest effects, followed by the CC. These shifts suggest that cognitive impairment may accentuate frailty‐related vulnerability in specific long‐range tracts (e.g., CR), whereas in cognitively normal adults, posterior regions (OL and PL) may show more prominent microstructural sensitivity to frailty. R1 and R2 were derived from SPGR and bSSFP steady‐state sequences with very short echo times, making them particularly sensitive to the fast‐relaxing water pool trapped between myelin bilayers. R1 primarily reflects differences in lipids and macromolecular content and overall tissue composition, while R2 captures interactions between water and its microenvironment, including myelin packing, axonal integrity, iron content, and microstructural organization. Because R1 is strongly influenced by lipid‐rich macromolecular content and myelin is one of the most lipid‐dense structures in the brain, the regional similarity between R1 and MWF associations is biologically plausible. However, reductions in R1 or R2 may also reflect axonal degeneration or other microstructural alterations and, therefore, should not be interpreted as exclusively reflecting demyelination. Moreover, although MWF is myelin‐specific, R1 and R2 are highly sensitive to myelin content and provide robust confirmation of the MWF findings. The subtle regional differences between R1/R2 and MWF may reflect additional aspects of WM microstructure, such as axonal degeneration, which could be explored in future multishell diffusion MRI studies (Alsameen et al. 2023; Gong et al. 2025). Notably, the CC and CR remained consistently affected across analyses, reflecting both their biological vulnerability and the higher reliability of quantitative MRI in large, coherent fiber bundles (e.g., reduced partial‐volume effects and higher signal‐to‐noise ratio). Our ROI‐specific analyses confirm that higher frailty is linked to lower levels of myelin content and broader microstructural alterations, with regional heterogeneity in effect size. Collectively, these findings indicate that while frailty exerts widespread effects on myelin and WM microstructure, the regional expression of these associations differs depending on cognitive status.

These regional vulnerability patterns align with prior neuroimaging and neuropathological literature. Lifespan relaxometry and diffusion MRI studies indicate that late myelinating WM tracts, such as the CC and CR, exhibit greater age‐related microstructural decline than earlier myelinating tracts, consistent with models of retrogenesis (Bartzokis et al. 2003; Bartzokis 2004; Barrick et al. 2010; Sexton et al. 2014; Arshad et al. 2016; Kiely et al. 2022). Age‐related reductions in WM integrity measured by DTI‐FA and other indices have been documented in both the CC and CR across adult cohorts (Mendez Colmenares et al. 2023). Moreover, voxel‐wise DTI analyses reveal widespread age effects in posterior regions including the posterior CR and splenium of the CC, supporting the notion that posterior pathways are sensitive to early microstructural changes (Bendlin et al. 2010; Ouyang et al. 2021; Mendez Colmenares et al. 2023).

### 4.4 Frailty and White Matter: Causality and Shared Mechanisms

A critical question arising from these findings is the directionality of the association between frailty and myelin/WM alterations. Our cross‐sectional data cannot establish causality; thus, it remains unclear whether demyelination and WM damage contribute to systemic frailty, whether frailty drives myelin/WM loss, or whether both reflect a common underlying biological process. One possibility is that myelin degradation impairs neural network efficiency, motor function (Akhonda et al. 2023; Faulkner et al. 2023; Gong, Faulkner, et al. 2024), and cognitive reserve (Gong et al. 2023; Gong, Bilgel, et al. 2024), thereby contributing to frailty. Conversely, systemic frailty, characterized by chronic inflammation, metabolic dysregulation, and reduced physiological resilience, could accelerate myelin and WM microstructural decline. A third scenario is that both phenomena are parallel manifestations of accelerated aging or cumulative physiological stress, reflecting shared molecular or vascular vulnerabilities. Notably, our observation that FI remains associated with myelin and WM measures even after accounting for age suggests that frailty captures aspects of biological vulnerability beyond chronological aging, supporting the notion of converging pathways. Future longitudinal and mechanistic studies are required to establish whether myelin and WM alterations are a cause, consequence, or co‐manifestation of systemic frailty.

### 4.5 Limitations and Future Directions

A major limitation of this study is its cross‐sectional design, which prevents inference about the temporal direction of associations. Although the BLSA is an ongoing longitudinal study, longitudinal multicomponent relaxometry data are currently available for only a limited number of participants and follow‐up visits. Additional follow‐up time and a larger number of repeated assessments will be required to enable adequately powered longitudinal modeling of myelin‐sensitive measures. As these data continue to accrue, future analyses will allow characterization of within‐individual trajectories of myelin change and their temporal relationship to frailty progression. Such longitudinal work will be essential to determine whether myelin alterations precede functional decline, arise as a consequence of systemic vulnerability, or reflect shared biological aging processes. Additionally, although the BLSA cohort is well characterized, it represents a relatively healthy volunteer sample, which may limit generalizability. Replication in larger, community‐based cohorts with a broader range of frailty severity will be important. Integrating molecular biomarkers with myelin‐sensitive imaging in longitudinal frameworks may further clarify mechanistic pathways linking systemic frailty and myelin integrity.

Furthermore, prior work has demonstrated that conventional mcDESPOT implementations may exhibit parameter identifiability and precision limitations under realistic signal‐to‐noise (SNR) conditions, particularly when intercompartmental exchange is incorporated into the model (Lankford and Does 2013; West et al. 2019). Importantly, for steady‐state SPGR and bSSFP acquisitions, intercompartmental exchange is biophysically expected to occur on time scales that are relevant to the observed MRI signal, and therefore represents a more complete description of the underlying tissue microstructure. However, explicitly modeling exchange substantially increases model complexity and leads to identifiability and numerical stability challenges under realistic SNR conditions, often resulting in parameter degeneracy and reduced precision (West et al. 2019). As a result, the BMC‐mcDESPOT implementation used here assumes negligible intercompartmental exchange to obtain stable and robust parameter estimates. This simplification is expected to introduce bias in the estimation of MWF and relaxation parameters, and therefore these metrics should be interpreted as model‐dependent estimates rather than direct or ground‐truth measures of myelin content or microstructure. While BMC‐mcDESPOT provides a practical and sensitive framework for in vivo myelin mapping, its estimates reflect a balance between biophysical realism and statistical identifiability. Notably, independent validation using transcriptomic and proteomic measures demonstrates that BMC‐mcDESPOT‐derived MWF tracks regional myelin‐associated molecular content in human brain tissue (Bae et al. 2026). Consequently, the consistency of findings across complementary metrics (MWF, R1, and R2) supports the robustness of the observed associations, despite these modeling assumptions. More broadly, mcDESPOT, like other quantitative MRI techniques, relies on idealized signal models that may deviate from in vivo tissue behavior. Received MRI signals can be influenced by multiple experimental and physiological factors, including diffusion, intercompartmental exchange, off‐resonance effects, magnetization transfer, J‐coupling, spin locking, internal gradients, and imperfect magnetization spoiling. The magnitude of these effects depends on tissue characteristics as well as pulse sequence design and acquisition parameters (e.g., TE, TR, flip angles, RF pulse shape, and gradient timing).

Although prior studies suggest that myelin content follows a nonlinear (e.g., quadratic) trajectory across the lifespan (Arshad et al. 2016; Kiely et al. 2022; Lo et al. 2025), our sample lacked dense representation in midlife (approximately 45–60 years), which may have limited sensitivity to detect such effects. Consistent with this, the quadratic age term approached significance but did not survive correction for multiple comparisons, and therefore should be interpreted with caution.

Finally, although the FI incorporates a broad set of health deficits across multiple physiological domains, including chronic conditions, functional limitations, weight loss, physical activity, and cognitive function, other factors such as systemic inflammatory biomarkers, socioeconomic status, and subclinical vascular risk are not explicitly modeled and may contribute to both frailty and myelin integrity. Because our regression models adjust only for age and sex, residual confounding cannot be fully excluded. Although all participants were scanned using the same MRI scanner and identical sequences, reducing scanner‐related variability, the cross‐sectional design and potential residual confounding limit causal inference, and future work incorporating additional covariates and larger, longitudinal imaging studies will be important for further clarification.

## 5 Conclusions

Higher frailty across the adult lifespan is associated with lower myelin content and WM integrity, as evidenced by widespread negative relationships between the FI and MWF, R1, and R2. These findings identify myelin‐related microstructural vulnerability as neural correlates of frailty. Myelin‐sensitive MRI may therefore provide a useful biomarker for detecting early brain vulnerability associated with multisystem aging.

## Author Contributions

M.B. and L.F. conceived and designed the study. M.B. performed the data analysis. T.T. contributed to the Frailty Index (FI) calculations. Z.G. Contributed to MRI data processing. All authors participated in writing, editing and revising the manuscript.

## Funding

This research was supported by the Intramural Research Program of the National Institutes of Health (NIH). The contributions of the NIH authors were made as part of their official duties as NIH federal employees, are in compliance with agency policy requirements, and are considered Works of the United States Government. However, the findings and conclusions presented in this paper are those of the authors and do not necessarily reflect the views of the NIH or the U.S. Department of Health and Human Services.

## Disclosure

Permission Statement: The authors affirm that all materials used in this study, including data, images, and figures, are original or have been appropriately cited. No third‐party content was used without permission.

## Ethics Statement

This study was conducted in accordance with the Declaration of Helsinki. All procedures were approved by the Institutional Review Board (IRB) of the National Institute on Aging (NIA). The study adhered to ethical guidelines for research involving human subjects.

## Consent

All participants provided written informed consent prior to participation.

## Conflicts of Interest

The authors declare no conflicts of interest.

## Data Availability

Data supporting the findings of this study are part of the Baltimore Longitudinal Study of Aging (BLSA), and access is governed by BLSA data‐sharing policies.
